# Phenotypic and Evolutionary Consequences of Social Behaviours: Interactions among Individuals Affect Direct Genetic Effects

**DOI:** 10.1371/journal.pone.0046273

**Published:** 2012-11-30

**Authors:** Barbora Trubenová, Reinmar Hager

**Affiliations:** Computational and Evolutionary Biology, Faculty of Life Sciences, University of Manchester, Manchester, United Kingdom; Centers for Disease Control and Prevention, United States of America

## Abstract

Traditional quantitative genetics assumes that an individual's phenotype is determined by both genetic and environmental factors. For many animals, part of the environment is social and provided by parents and other interacting partners. When expression of genes in social partners affects trait expression in a focal individual, indirect genetic effects occur. In this study, we explore the effects of indirect genetic effects on the magnitude and range of phenotypic values in a focal individual in a multi-member model analyzing three possible classes of interactions between individuals. We show that social interactions may not only cause indirect genetic effects but can also modify direct genetic effects. Furthermore, we demonstrate that both direct and indirect genetic effects substantially alter the range of phenotypic values, particularly when a focal trait can influence its own expression via interactions with traits in other individuals. We derive a function predicting the relative importance of direct versus indirect genetic effects. Our model reveals that both direct and indirect genetic effects can depend to a large extent on both group size and interaction strength, altering group mean phenotype and variance. This may lead to scenarios where between group variation is much higher than within group variation despite similar underlying genetic properties, potentially affecting the level of selection. Our analysis highlights key properties of indirect genetic effects with important consequences for trait evolution, the level of selection and potentially speciation.

## Introduction

Complex social interactions are widespread among animals and are of considerable interest to both behavioural ecologists [1]–[3] and quantitative geneticists [4]–[6]. Understanding the role of interactions among individuals for phenotypic variation and fitness is crucial for the study of such diverse areas as behavioural ecology, where IGEs are implicated in the evolution of cooperation and social dominance [2], [7]–[11] and agriculture (inform breeding design, reduction of competition) [12], [13]. From an evolutionary perspective, any interaction or behaviour can be regarded as social whenever it influences not only the fitness of the actor, but also the fitness of other individuals [14]. For example, cooperation, altruism, but also aggression and spite are social behaviours, and traits underlying these behaviours are influenced by interactions with conspecifics [15]–[18].

Standard quantitative genetics theory assumes that the phenotype, or trait values of an individual, are affected by its genes and the environment. Environmental effects are usually considered to have no genetic basis, and therefore be non-heritable. However, every individual living in a social environment is affected by the social behaviours of conspecifics, which is partly given by their genotypes. The environment provided by conspecifics is often the most important component of the environment experienced by individuals, frequently having profound effects on trait expression and fitness [19].

The influence of the genotypes of other individuals on the phenotype of a focal individual is referred to as indirect genetic effect (IGE) [4], [8], [20]–[25] or associative effect [26]–[28], because a social partner's genes influence the trait indirectly - they are expressed in interacting individuals, not in the individual whose phenotype is observed.

The idea that the phenotype of an individual is affected not only by the genes it carries, but also by the genes of other individuals was proposed already by Hamilton [7] in his theory of neighbour modulated fitness. Further development of this concept was strongly linked with research in the field of parental care [4], [20], [29]. Therefore, IGEs are often illustrated by maternal effects [21], [30], where the phenotype of the mother influences expression of traits in her offspring (for example body size) [31], [32]. Maternal effects result in IGEs whenever the traits contributing to the environment provided by the mother are heritable. Maternal genetic effects often account for as much as half of the variance in characters expressed early in life [21].

However, social interactions among individuals are ubiquitous and IGEs are not limited to interactions among relatives [33]. Often, unrelated individuals interact and these interactions may strongly influence their respective phenotypes [34]. Behaviours such as aggression, cooperation and courtship are common examples of such interactions [18], [27], [35], [36]. Even the behaviour of primitive organisms, such as bacteria and amoeba, is affected by genes expressed in other individuals [37], [38].

There have been very few studies of IGEs within social groups [39]. In *Drosophila melanogaster*, the genotypic composition of social groups (single versus mixed genotypes) was shown to affect expression of clock genes, genes for pheromonal profile on the cuticle and mating frequency [40]. Several recent empirical studies have mapped the quantitative trait loci (QTL) for maternal genetic effects and other IGEs [34], [41]–[44], shedding light on the genetic architecture underlying IGEs.

Unlike effects of the physical environment, social influences have both an environmental and genetic component. Because of this genetic (heritable) component, social influences are subject to selection and can evolve, while the environmental component may also act as a selection factor. By affecting the strength of selective pressure and changing the expected genotype-phenotype relationship, IGEs can change the speed and direction of evolution [4], [20], [36]. Thus, IGEs and social interactions play an important role in trait evolution.

Models of IGEs are traditionally formulated in two different ways: the trait-based approach developed by Moore [20] focuses on how a phenotype of a focal individual is influenced by specific traits in social partners. The second approach, developed in a series of papers by Griffing [45]–[48], was first adopted by Muir [26] and later by Bijma and colleagues [12], [13], [49]–[51], looks at the total genetic variance and its partitioning into two independent components. The direct variance component arises from an individual's own genotype while the indirect component is caused by interactions among individuals [23]. The second approach is favoured by empiricists because the methods of estimating variance components are relatively straightforward. A detailed comparison of both frameworks was carried out by McGlothlin and Brodie III [23], who demonstrated the compatibility of both approaches.

Here, we provide a detailed analysis of IGEs and their dependence on key parameters: the strength of interaction between individuals, group size and population genotypic characteristics. Unlike Moore [20], we consider interactions of multiple individuals and demonstrate how group size can affect the strength of IGEs, the mean phenotype and its variance. Furthermore, we investigate the effect of interactions on within and between group variation and discuss the possible implication for multilevel selection. In contrast to previous models, we formally distinguish two separate consequences of social interactions: first the indirect genetic effect and, second, the change in the direct genetic effect. Previous trait based models did not distinguish between these two separate effects [20], [36], while variance-based models [12], [13] did not explore the effect of interactions on direct genetic effects. However, we can show that both direct and IGEs may reach extreme values, either cancelling each other (if the effects are of opposite sign) or increasing effects on phenotypic values (if of the same sign). We further develop our model in a way suitable for agent-based modelling, commonly used to study social evolution [52]–[55], and use simulations to illustrate our results.

## Results

### The model

We begin by generalizing Moore et al. 's model [20] for the case with N individuals
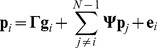
(1)where 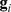 (genotype) denotes a column vector of genotypic values of an individual's genes, 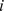, 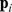 (phenotype) is a column vector of corresponding trait values (same as 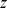 in [20]), and 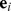 is a vector of environmental components acting on the i-th individual. As we want to investigate the potential role of social interactions on direct and indirect genetic effects, we will not consider environmental effects other than those caused by social interactions in the following analysis. Therefore, 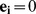 in our model. However, environmental influences can easily be incorporated into all calculations, by replacing 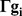 (the genetic component) by 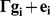 in all following equations (see Methods).

The first term represents direct genetic effects (DGE), with matrix 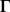 mediating the translation of an individual's own genotype 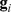 into its trait values. For the simplest models, where every trait is encoded by a single gene, this matrix is square and diagonal. However, in a more realistic scenario multiple genes affect the same trait or the same gene affects more than one trait (pleiotropy). These effects can be incorporated into the model by populating matrix 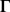, with size 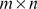, where 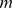 is the number of traits under investigation and 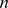 is the number of genes involved.

The second term represents associative effects [13] that may be interpreted as heritable environmental effects provided by social partners of the focal individual [4]. Matrix 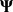 is a square (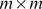) interaction matrix [4], [20], [36], in which 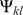 defines the effect of the partner's trait 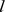 on the trait 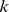 of the focal individual. If 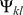 equals 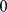, there is no effect. If it is negative, a higher expression of the partner's trait 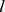 lowers the expression of the focal individual's trait 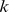. Positive 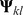 means that the expression of trait 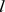 enhances the expression of trait 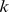 in the focal individual. 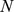 denotes the number of individuals in the group, 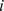 the focal individual and 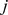 all other individuals in the group.

To investigate the dependence of the focal individual's phenotype on the genotypes of its social partners, it is necessary to separate the focal individual's genes from those of its conspecifics and rewrite equation 1 as follows
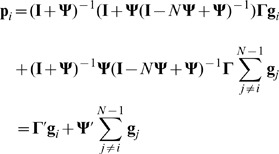
(2)where 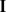 is an identity matrix and
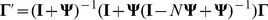
(3)

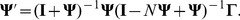
(4)


**Figure 1:**
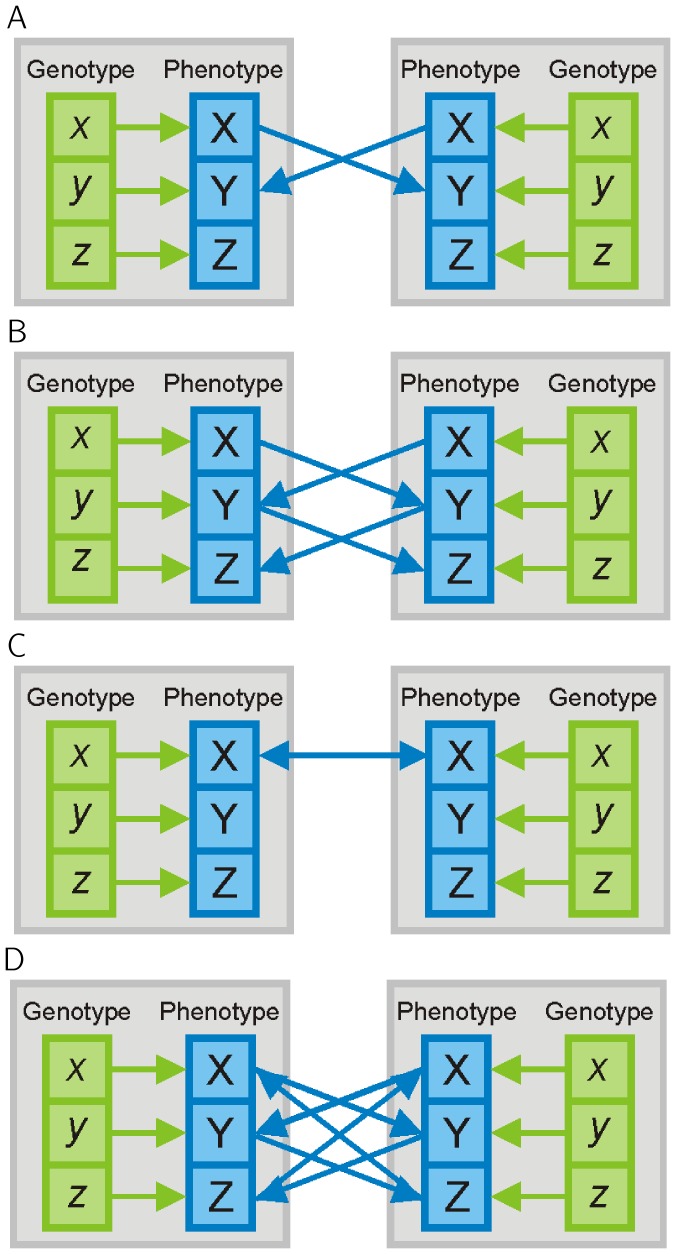
Different classes of interactions. (A) Example of interactions without feedback. (B) Example of interactions with feedback at the individual level. (C, D) Example of interactions with feedback at the trait level: (C) one trait affecting itself. (D) three traits involved in feedback to themselves via multiple interactions. Boxes represent individuals, blue squares represent phenotypic traits, green squares represent genes. Blue arrows represent matrix 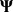, green arrows represent matrix 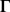.

The first term of equation 2 (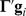) focuses only on the focal individual's genes, and therefore represents DGEs [4]. It should be noted that the term 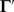 specifying the dependence of an individual's phenotype on its genotype is more complicated than in a case without any interaction (when 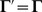). This complication arises from the potential for feedback loops that may occur when a trait affects itself in a social partner [6], [20], [23] or when a trait affects some other trait in the same individual via interactions with traits expressed in conspecifics. In such a case, the phenotypic value of a particular trait is not only given by genes directly underlying this trait and by genes expressed in conspecifics, but also indirectly by other genes expressed in the focal individual (via the interaction with other individuals). For example, if a trait X affects trait Y in conspecifics, while trait Y affects trait Z, the phenotypic value of Z is given by an individual's own genes for Z, as well as X (see Figure 1 B). This suggests that interactions of individuals do not only change their phenotypes due to IGEs, but they can also change the way the genotype of the focal individual is translated into its own phenotype (direct effect). A similar effect was described as 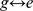 interdependence: organisms change the environment around them, which in turn changes the expression of their genes, for instance via stress, and are especially common in the case of social interactions [56]. In our case, 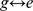 interdependence is represented by the coefficient 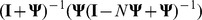 that alters gene expression from 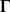 to 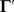.

The second term 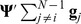 in equation 2 represents IGEs. IGEs depend only on the genes of interacting individuals and not on the genes of the focal individual. While it is obvious that the IGEs are the product of matrix 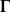 and the interaction matrix 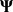, it is not obvious why the terms 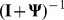 and 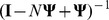 appear. These terms are present due to the possibility of feedback interactions, i.e. when a trait in the focal individual changes a trait in interacting individuals, which, in turn, changes the same or a different trait in the focal individual. Indeed, 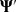 simplifies to 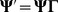 in the absence of feedback interactions. Note, that in order to infer equation 2 we assume that all interactions are symmetric.

In equation 2, 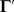 can be interpreted as the relative effect of an individual's (own) genes for its phenotype compared with the effects of genes in conspecifics for the phenotype of the focal individual (given by 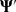). There is a simple relationship between the relative importance of an individual's own genes and the importance of genes in conspecifics, given as:
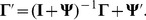
(5)


### Three classes of interactions

As stated above, some interactions may contain a feedback loop that occurs when one trait affects the same trait in a social partner or when it alters the expression of a different trait in the focal individual via interactions with social partners [6], [20], [23], [26]. Our analysis shows that interactions involving feedback loops have different properties from those without such feedback (Figure 1 A). Furthermore, interactions with a feedback loop at the individual level, but not the trait level (Figure 1 B), yield different outcomes from interactions when the feedback loop points back to the same trait (Figure 1 C–D).

#### Interactions without feedback

The first, simplest class involves situations, in which a trait in a focal individual influences some other trait in its social partners, however, these affected traits do not alter the expression of any trait in the focal individual (Figure 1 A). A common example of such a case is the effect of body size on aggressive behaviour, as individuals often mediate their aggressiveness according to the body size of their opponents [57]–[59]. The first type of interaction between two individuals described in Moore at al. [20] belongs into this class.

**Figure 2:**
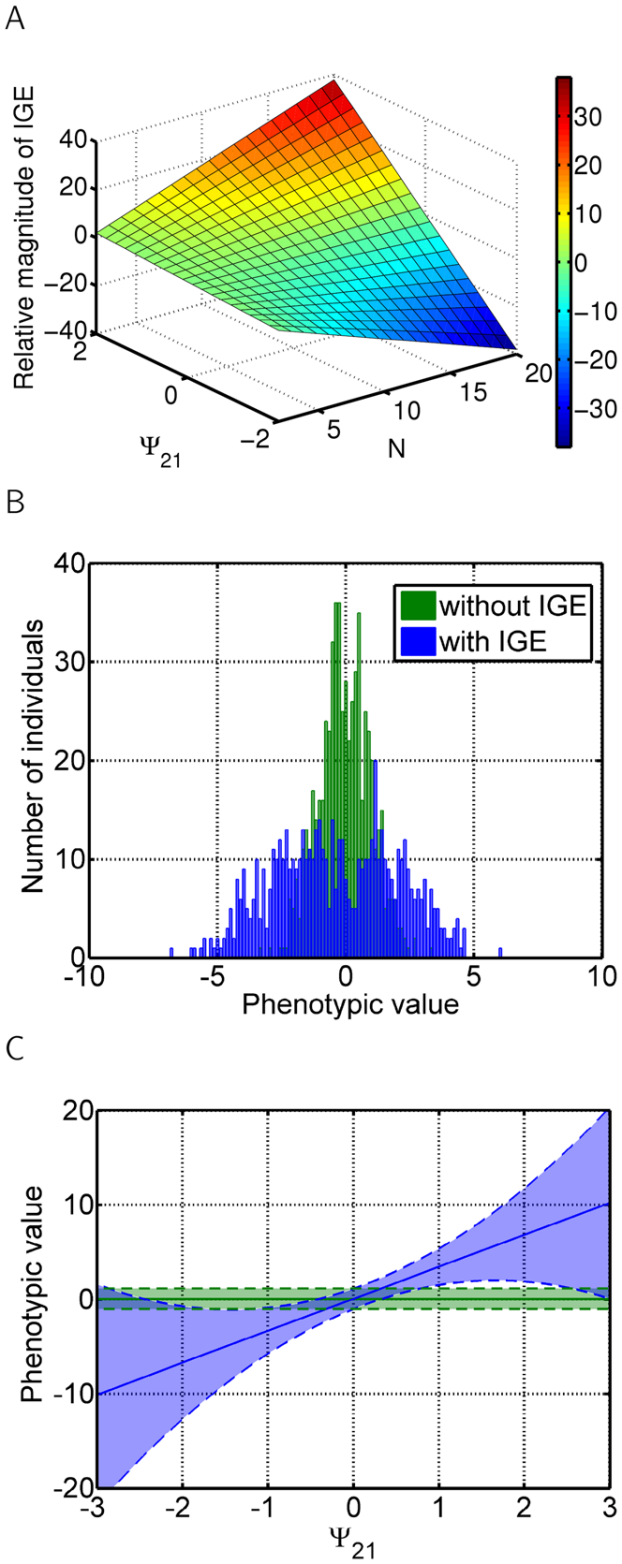
Interactions without feedback. Indirect genetic effects of gene x on trait Y (see Figure 1 A), with trait X affecting trait Y. (A) Relative magnitude of indirect genetic effect, as described in equation 6, with the strength of the interaction 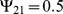. (B) Histogram of the phenotypic values across population. Green - without IGEs, blue - with IGEs. Strength of the interaction set to 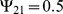. (C) Mean phenotypic value (solid line) plus and minus variance (dashed lines). Blue - mean phenotype and its variance if IGEs are considered. Green - no IGEs are considered. Both (B) and (C) are results of the simulations of a group of 30 individuals.

It is easily seen whether or not a situation belongs to this class - only unaltered traits may affect other traits, otherwise feedback occurs. Formally, the condition 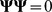 must be fulfilled. For this class of interaction, there is no 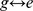 interdependence and equation 2 can be simplified to
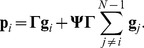
(6)Here, the IGE magnitude depends linearly on the strength of interactions (elements of matrix 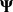). Therefore, the stronger the effect of the other's phenotype on the phenotype of the focal individual, the stronger the indirect genetic effect, as illustrated in Figure 2 A. Direct genetic effects are not altered by these interactions. However, as phenotypes of all individuals are changed by IGEs, there is a change in the distribution of phenotypic values of the affected trait (see Figure 2 B).

The mean phenotype is given by
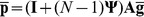
(7)and depends linearly on group size 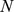 and the strength of interactions, depicted in Figure 2 C. Here, 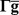 defines the average phenotype when no interaction occurs, while 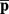 is the average phenotype when interactions do occur.

To obtain the relationship between an individual's phenotype and the group mean phenotype, we have to express an individual's genotype as its deviation from the mean genotype
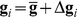
(8)and substitute 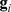 in equation 6. An individual's phenotype can thus be expressed as
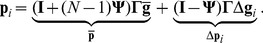
(9)


The first term corresponds to the mean phenotype, while the second term represents an individual's deviation from the mean phenotype. Note that an individual's deviation from the mean phenotype is linearly dependent only on the strength of the interaction and the deviation from mean genotype but not on the number of individuals.

Phenotypic (within group) variance for each trait can be written as
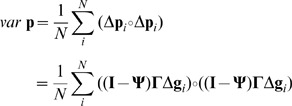
(10)where 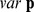 is a vector of variances of all traits (NB. 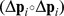 is the Hadamard product of the vectors). Therefore, the variance of a given trait is not only given by the genetic variation of genes directly underlying the trait but also by the variation found in all genes affecting this trait.

#### Interactions with feedback on the level of individuals

The second class comprises situations, in which a trait in a focal individual (trait X) affects a different trait in its social partners (e.g. trait Y), which in turn influences a trait Z, different from both X and Y in the focal individual. This scenario describes two partial interactions: trait X affecting trait Y and trait Y affecting trait Z. However, no trait affects itself by any set of partial interactions (see Figure 1 B). In other words, a feedback loop does return back to the same individual, but not to the same trait. An example of such a situation may be scent marks that advertise territoriality to competitors, which may change the competitor's behaviour toward the signaller (and hence affect the latter's phenotype) e.g. in rodents [60]. Another possibility is the degree of cooperation among individuals. In such cases, an individual's scent induces cooperative behaviour in more closely related individuals, which in turn may affect body size (trait Z) of the focal individual. Note, that interactions in this class are distinct from those described in Moore et al. [20].

**Figure 3:**
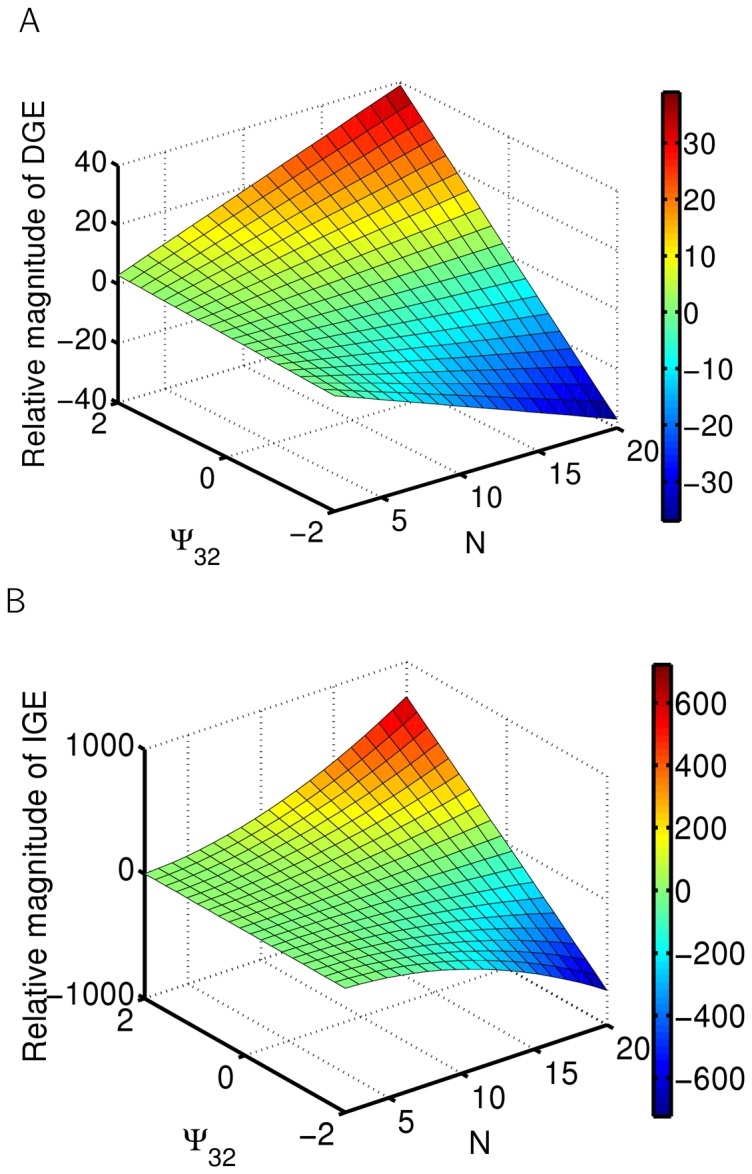
Interactions with feedback at the individual level: direct and indirect genetic effects. Dependence of the direct (A) and indirect (B) genetic effect on the strength of the interaction 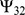 (trait Y acting on trait Z) and group size 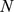. Visualization of DGE and IGE of genes *x* and *y* acting on Z, as described in equation 2. Interaction, as depicted in Figure 1 B, with the interaction strength of X acting on Y set to 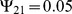.

For this class of interaction, equation 2 cannot be simplified any more and IGEs depend linearly on all partial interactions involved. The effect of an individual's own genotype on its phenotype (direct genetic effect) is altered as well (see Figure 3 A). For example, an IGE acting on trait Z depends linearly on both coefficients of interaction of trait X acting on trait Y and trait Y acting on the trait Z (Figure 1 B). However, an IGE on trait Z depends now quadratically on the number of individuals: although linearly dependent on each partial interaction, trait Z is now affected by two subsequent interactions, as depicted in Figure 3 B (as 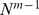 where 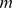 is the number of traits involved).

From equation 2, the mean phenotype 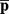 can be expressed as
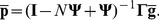
(11)To obtain the relationship between an individual and the mean phenotype, we substitute equation 8 in equation 2. An individual's phenotype can then be written as
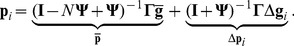
(12)As 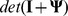 and 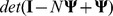 are non zero for any value of 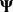 in this class, it is always possible to find inverse matrices of both terms. Therefore, phenotypic values can always be predicted using equation 12 for any combination of parameter values.

The within group phenotypic variance of each trait is given by
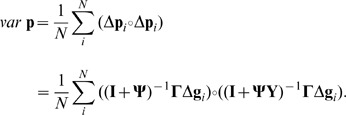
(13)


#### Interactions with feedback on the trait level

The last class contains situations, in which a particular trait affects its own expression by interacting with other individuals. Both types of interactions described in Moore et al. [20] belong to this class of interactions. An example of such a case is aggression, where aggressive behaviour of one individual induces aggressiveness of its opponent, which in turn may increase aggressive behaviour of the first individual, e.g. in primate groups [61] (Figure 1 C). It is not necessary that only one trait is involved - two distinct traits can reciprocally affect each other, or even more traits can be involved (Figure 1 D). The key point is that the expression of a particular trait feeds back to alter itself.

**Figure 4:**
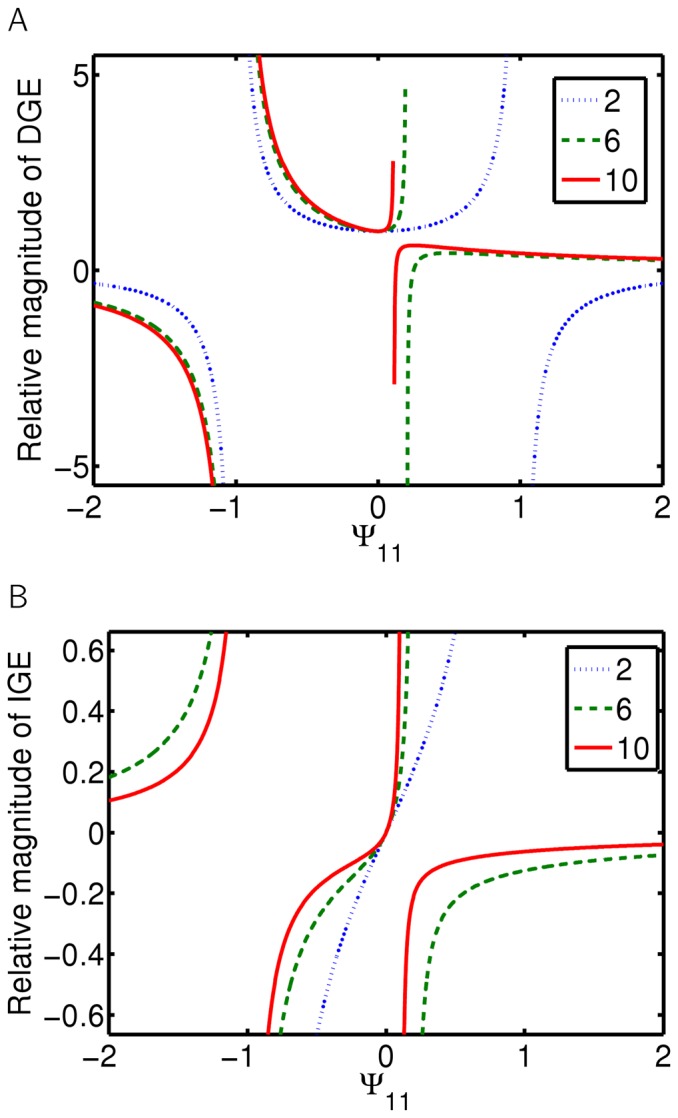
Interactions with feedback at the trait level: direct and indirect genetic effects. Relative magnitude of direct (A) and indirect (B) genetic effects and their dependence on the strength of the interaction, for different numbers of individuals (two, six and ten individuals). Interaction as depicted in Figure 1 C, where 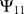 is the strength of the interaction between trait X of the focal individual and the same trait in its social partner.

Such feedback introduces a possibility of increasing trait values to infinity, as depicted in Figure 4. Indeed, unlike in the previous class, the terms 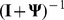 and 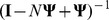 can introduce undefined points, when 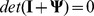 or 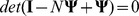. When elements of matrix 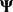 (specifying the strength of the interactions) are close to these points, DGEs and IGEs reach extreme values. This phenomenon was observed by Moore et al. [20], however, since they dealt only with dyadic interactions, several aspects of this phenomenon could not be explored - for example the dependence of one of the undefined points (and hence extreme phenotypes) on group size. The dependence on group size may be very important for many aspects of behavioural biology such as group size and stability. For instance, if the interaction strength is 0.145, the level of aggressiveness may be acceptable for groups with 5 members, but the arrival of an even mildly aggressive sixth member would increase the overall aggression by more than 5 times.

Note that both direct and indirect genetic effects may reach extreme values under the above conditions as the effects of 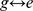 interdependence can be very strong. If the direct and indirect genetic effects are of the same sign, they may cause extreme phenotypes. However, if they are of opposite signs and of relatively similar (though extreme) values, they cancel each other and may lead to a phenotype similar to the case of no interaction.

Individual and mean phenotypes, as well as variances are defined as in the case of feedback at the level of the individual (equations 12, 11 and 13, respectively). When considering equation 12, the undefined points are separated - one is responsible for extremes of the mean phenotype and one for extreme deviations of individual phenotypes from the mean phenotype. Therefore, an individual can have an extreme phenotype due to two independent factors - either the individual phenotype deviates extremely from a relatively normal mean phenotype, or the mean group phenotype is extreme while the individual's phenotype deviation is relatively small.

**Figure 5:**
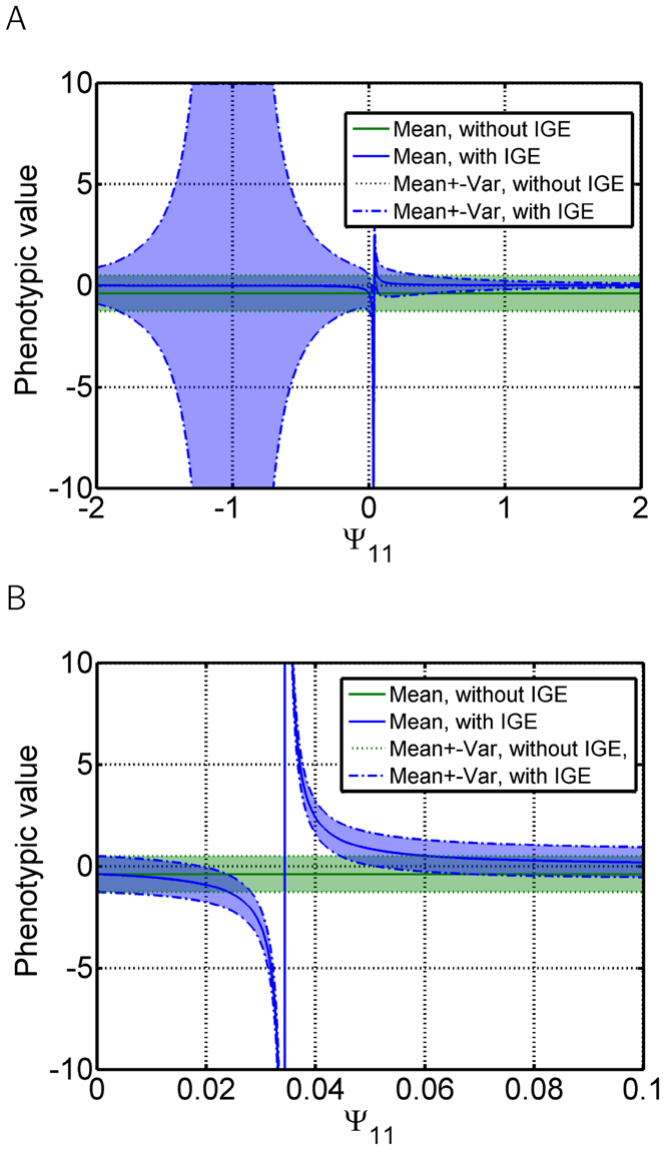
Interactions with feedback at the trait level - mean and variance. Dependence of the mean group phenotypic value and its variance on the interaction strength 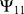, when one trait affects itself in other individuals (as depicted in Figure 1 C). Mean and variance (A) around 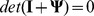, (B) around 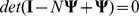. Blue - mean phenotype and its variance if IGEs are considered. Green - no IGEs are considered. Simulation of 30 interacting individuals.

The former occurs when values of 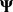 are close to 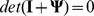. In such a case, all phenotypes are distributed around a normal mean with extremely high variance (Figure 5 A). Thus, differences among individuals within groups are much higher than differences between groups. This term does not depend on the number of individuals in the group, just on the strength of the interactions and genetic deviations.

**Figure 6:**
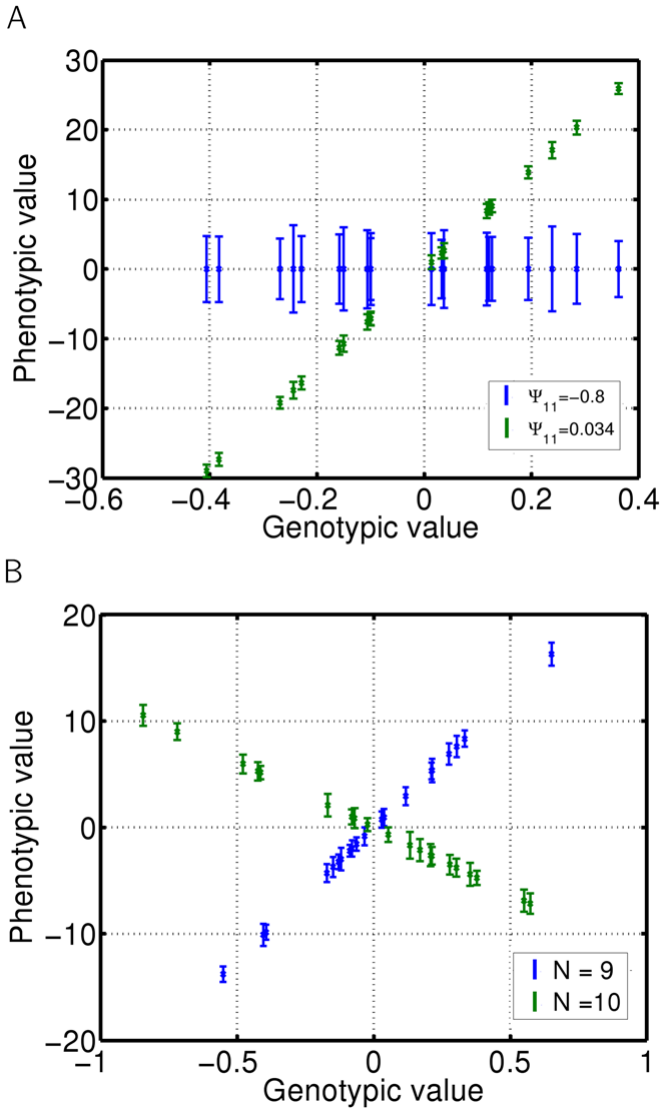
Inter and intragroup phenotypic variance. Mean phenotypic value and variance of each group are calculated and plotted against mean genotypic value of the group. Errorbars represent intragroup variance. Simulation of a scenario when a given trait in a focal individual affects the same trait in other individuals, as shown in Figure 1 (A). Simulation of 20 groups of 30 individuals. Blue - interaction strength 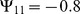, green - interaction strength 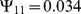. (B) Simulation of 20 groups of 9 (blue) or 10 (green) individuals; interaction strength 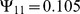.

On the other hand, when 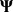 is close to 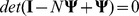, mean group phenotypes reach extreme values and even small variances in the mean genotype of the group may cause drastic changes to the mean phenotype. However, the phenotypes of all individuals in each group are distributed around the mean phenotype with relatively small variance (Figure 5 B). Thus, intergroup variation is much higher than intragroup variation. In this scenario, small differences in mean genotypes between groups are translated into large differences between the mean phenotypes of such groups (Figure 6 B). Therefore, if a trait affecting fitness is modified in such a way, individual fitness may strongly depend on the properties of the group, such as genotypic mean and variation among group members and group size. Since all individuals are similar to each other in each of these groups, such low intragroup and large intergroup variance may thus lead to selection on the level of groups and potentially speciation. Note that the range of the interaction strength 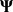, leading to large intergroup variance, is much smaller than the range of values leading to large intragroup variance (Figure 5 A and B). The effect of interaction strength on intra and intergroup variance is shown in Figure 6 A.

The value of the mean phenotype depends on the strength of the interaction, the mean genotype, and the number of interacting individuals. This means that the same type of interaction may have very different effects on phenotypes of individuals belonging to two differently sized groups. This applies, for example, to groups of nine and ten individuals when 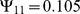. In this case, the interaction effect on the mean group phenotype in groups of ten individuals is much stronger than the effect in groups of nine individuals, as illustrated in Figure 6 B. The variance within groups is relatively small and similar for groups with nine and ten members. Note, that the value of interaction strength is smaller than 1/(N-1). This means that the relative influence of all the other individuals is still smaller than individual's own genotype, which is a reasonable assumption. Extreme phenotypic values are not caused by strong interactions.

The effect of group size on IGEs has been investigated mostly focusing on of the dilution effect in larger groups [24], [27], [36]. Dilution is a decrease of IGEs with group size [24], for instance because individuals interact less often. Without such dilution, individuals in larger groups should experience stronger effects, as more individuals contribute to the IGEs. While this may apply to the first two classes of interaction (where the effects depend linearly on group size) it does not for the third class. When interactions feed back on themselves, the dependence on group size is no longer linear.

### Fitness and selection

When a fitness of an individual is affected by a phenotypic trait of conspecifics, social selection is said to occur. However, interacting individuals may affect each other's fitness indirectly as well, via IGEs - affecting those traits of the focal individual that are important for fitness.

If we assume 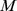 groups of 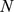 interacting individuals, the relative fitness of each individual can be defined as
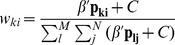
(14)where 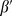 is a column vector describing the importance of an individual's own traits for its own fitness and C is a constant. Note that the mean fitness of the population is defined as 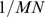 as opposed to 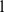 as often used elsewhere, so the sum of the fitness of all individuals across the population is 1. We use this value for the convenience of the calculations and simulations.

If we normalize values so that 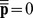 and use equation 2 for the phenotype of a focal individual, its relative fitness can be written as
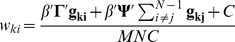
(15)or
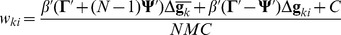
(16)where 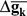 denotes the difference between the mean genotype of the 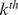 group and mean population genotype, while 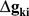 is a difference between individuals own genotype and mean genotype of its group.

#### Response to selection

To understand the effect of interactions on trait evolution, we have to investigate their effects on the response to selection. The response to selection 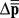 is defined as a difference between the mean phenotypic value of offspring and the mean phenotypic value of the parental generation, and can be calculated using Price's equation:
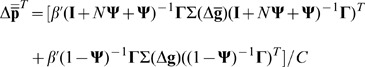
(17)where 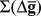 is a variance-covariance matrix of mean group phenotypic values of each trait (between group variance-covariance), and 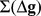 is a variance-covariance matrix of traits within the group (within group variance-covariance).

**Figure 7:**
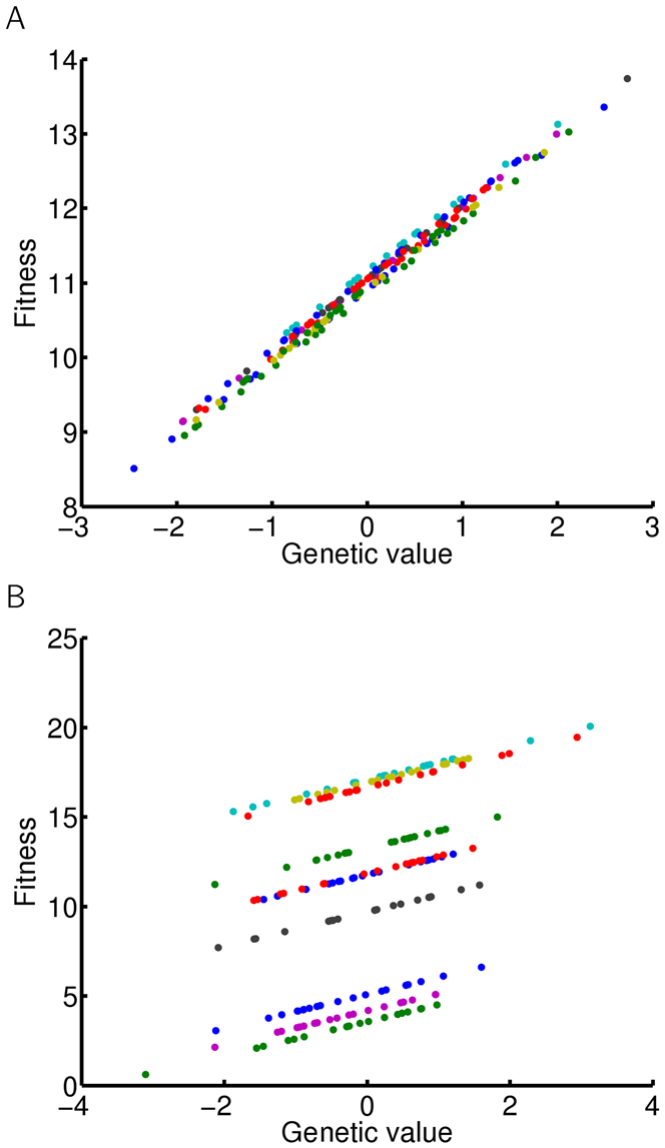
Inter and intragroup variance in fitness. Simulations of 10 groups of 20 interacting individuals; a given trait in a focal individual affects the same trait in other individuals (shown in Figure 1 C). Different colours represent individuals in different groups. (A) The value of 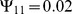. Within group variance is high, but between group variance is low. (B) The value of 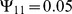. Within group variance is low, but between group variance is high.

Equation 17 shows that the response of selection depends not only on the overal genotypic variance of the population, but on the intragroup and intergroup genotypic variances as well. The strength of the indirect genetic effect given by 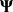 is a key factor in determining which of these variances becomes more important for the response to selection. If the strength of interaction is close to the value where 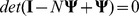, small differences between genotypic distributions of individual groups may translate into substantial differences in the fitness of groups. In such a case, group properties are much more important for individual's fitness, and the selection at the level of groups becomes much stronger than the selection at the level of individuals (Figure 7).

Furthermore, interaction strength 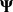 and the variance between groups and within groups determine whether the response to selection is positive or negative. When within group variance is smaller than between group variance, even traits that are non beneficial to the focal individual, but beneficial to the group, may create a positive response to selection, similar to the effect of social selection. This can be interpreted in terms of Hamilton's rule [62]: term 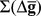 describes the relatedness between groups, while 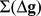 is the relatedness between individuals within each group. In this case, smaller variance within groups may be interpreted as higher relatedness in each group, while costs and benefits are given by the strength of interaction 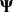 and the selection gradients 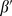.

## Discussion

The key result of our model is a detailed description and analysis of extreme phenotypes caused by feedback when IGEs occur. We show that DGEs can be drastically altered by social interactions. This profoundly influences the relative importance of an individual's genotype for its own fitness when compared to genes of its conspecifics. Furthermore, we can demonstrate that even small differences in the mean genotypes between groups may lead to large phenotypic and fitness differences between groups. This may lead to selection at the level of groups and potentially speciation, especially when considering traits involved in mate choice. Finally, social interaction effects may depend to a large extent on the number of interacting individuals, in a non-linear way.

We advance on previous models by investigating interaction effects among multiple individuals on their phenotypes. Specifically, we can predict the possible range of phenotypic values as well as their dependence on group size and genetic composition of the group. Thus, our predictions can be directly tested in systems that allow manipulation of group size and genetic composition of groups, as well as inter- and intra-group genetic variance (e.g. mixed litters in genetically variable mice [63]). Furthermore, we derive equations that can easily be incorporated into agent based models, e.g. to simulate more complex evolutionary scenarios for which no analytical solution can be derived.

Our analysis has shown that the phenotype of a focal individual is not only affected by IGEs, but importantly also by changing the dependence of an individual's phenotype on its own genotype (direct genetic effect). This phenomenon is akin to 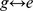 interdependence: organisms change the environment around them, which in turn changes the expression of their genes, for instance via stress [56]. Niche construction is an example where genotypes (i.e. individuals) modify their environment, which in turn affects the selective pressure on these genotypes [64], [65]. Clearly, when considering the social environment, 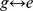 interdependences are especially common [56].

### Types of interactions

In our analysis, we distinguish between three classes of interactions that fully describe all possible ways in which IGEs can manifest. In the simplest class of interaction where no feedback occurs, IGEs depend linearly on group size and the strength of interactions. The stronger the interaction or the more individuals are in the group, the stronger the IGEs. However, this assumes that interaction strength does not depend on group size itself, which is not necessarily true. For example, the effect of interactions among particular individuals may be less intense in larger groups [24], where individuals interact less often. This may lead to a decrease in interaction strength (‘dilution’ - sensu [24]).

The second class comprises of situations, where a feedback loop exists to the same individual, but not to the same trait. An example of such a situation is cooperation based on kin recognition, when the presence of a specific trait increases cooperation (e.g. a green beard [66]), which in turn may affect body size (e.g. through better resource exploitation). Unlike the first class, IGEs will depend non-linearly on the number of interacting individuals 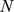 and on the number of traits 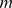 involved in the feedback loop (specifically, 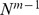).

On the other hand, interactions that feed back to the same trait, resulting in a trait affecting itself, represent our third class of interaction. Moore and colleagues showed that for interactions involving feedback, IGEs may reach extreme values as interaction strength (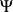) approaches 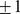 [20]. We generalized their findings and showed that both direct and indirect genetic effects are undefined when 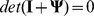 or 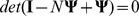. When the strength of interaction 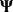 reaches values that satisfy one of these two conditions, 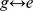 interdependence is very strong and both direct and indirect genetic effects may reach extreme values (extreme phenotypes). When an individual genotype is expressed as the mean group genotype plus the deviation from this mean, we can separate the above two conditions. The separation of these two conditions allows for comparisons of the importance of an individual's own genotype for the expression of its own phenotype relative to properties of its group (e.g. mean genotype). The first condition, (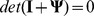), refers to an individual's deviation from the group mean phenotype, while the second condition, (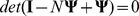), refers to the mean phenotype of a group. Our results suggest that, in a narrow interval close to 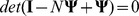, group properties become more important for an individual's phenotype than its own genes, with potential implications for the level of selection. Moore and colleagues observed that phenotypic trait values are undefined when 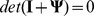 [20]. As they dealt only with dyadic interactions, the dependence of the second undefined point on the number of interacting individuals was not discussed. However, the dependence on group size may be very important for many aspects of behavioural and evolutionary biology, especially group size and group stability.

### Evolutionary consequences

From an evolutionary point of view, if 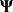 is close to 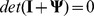, an individual's (genotypic) deviation from the mean genotype becomes much more important for its phenotype than its own genotype. Thus, if a trait is related to fitness, individual differences in fitness will be greater within groups than between groups (see Figure 7 A). In such cases, selection at the level of individuals may be very strong. However, if the strength of interaction is close to the second undefined point 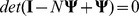, all phenotypes in the interacting group are sensitive to small changes such as the number of individuals or the mean genotype. All individuals in the group will have similar trait values with only small variation, while small intergroup differences in the mean genotype will be enhanced, and may cause large intergroup variation in phenotypic values. Again, if the trait is related to fitness, group properties such as mean genotype or the number of interacting individuals may lead to individuals within a group having higher fitness than individuals in a different group even if they have similar genotypes across groups (Figure 7 B). This may therefore create selection pressure at the group level, similar to the effect of social selection.

When interactions feed back on themselves, the dependence of IGEs on group size is no longer linear. Even lowering the number of interacting individuals while keeping the strength of interaction constant may lead to much stronger effects. If the trait in question (e.g. aggression) impacts on group stability [67], [68], the consequences of feedback interactions may affect group stability.

Our study highlights the importance of IGEs for trait evolution and shows the mechanisms by which IGEs manifest. Scenarios where IGEs and group properties become more important for trait evolution than DGEs and the potential for the occurrence of extreme phenotypes further suggests that IGEs may need to be considered for their role in speciation but certainly when quantifying community interactions [69] and their evolutionary consequences.

## Methods

We begin by generalizing Moore et al. 's model [20] for the case with N individuals
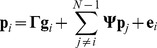
(18)In our model, 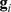 (genotype) denotes a column vector of genes of an individual 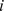, 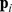 (phenotype) is a column vector of corresponding traits (same as **Z** in [20]). Matrix 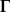 mediates the translation of individual's own genes 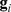 into its trait values. Matrix 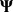 is an interaction matrix, where 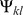 defines the effect of the partner's trait 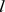 on the trait 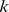 of the focal individual. For simplification, we do not consider environmental effects other than those caused by social interactions.

We can rewrite equation 18 as
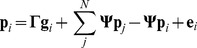
(19)Summation over all individuals in a group yields
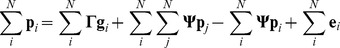


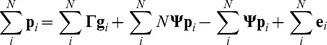


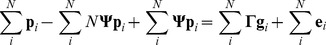
(20)Now, we can express the sum of all phenotypic values as
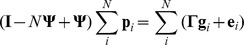


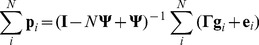
(21)A substitution of equation 21 into equation 19, we are able to express the phenotype of the focal individual as a function of genotypes of all group members
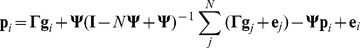


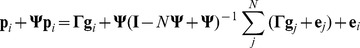


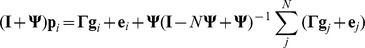


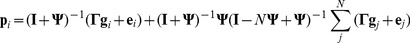
(22)To separate direct genetic effects (effect of individuals own genotype) from IGEs (effect of genotypes of other individuals), we have to subtract the effects of the genes of the focal individual from the second part of the equation 22.
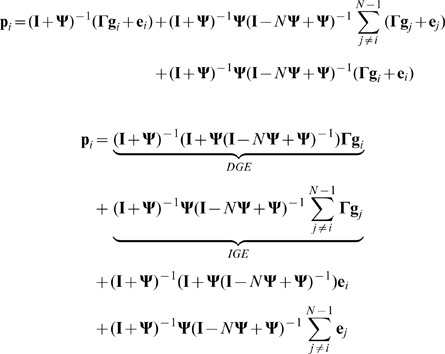
(23)Now, the first term of equation 23 depends only on the focal individual's genotype (i.e. DGE) while the second term depends only on the social partners' genotypes (i.e. IGE).

### Simulations

We analysed the role of interaction strength and number of individuals for both direct and indirect genetic effects in our model. Unlike previous frameworks, our model was developed with agent based modelling in mind. Equations were derived for individuals, therefore can be directly used in agent based models for calculations of an individual's phenotype, when genotypes are known.

To illustrate the interaction strength effect on intragroup and intergroup phenotypic variance, we simulated 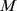 groups of 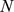 individuals (Figure 2 C, D; Figures 5, 6 and 7). All simulations in this study were carried out in Matlab R2010a.

We assume that each individual is haploid and has three genes and three traits. Groups were created by assigning each gene for each individual a random value sampled from a standard normal distribution. Then, the mean genotype of the whole population was calculated and subtracted from the genotype of each individual, thus the population mean genetic value of each gene was set to 0.

Figures 2 A, 3 and 4 were created using equation 2.

Matrix 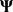 (3×3) was populated to describe a given interaction (see Table 1). We calculated phenotypes for all individuals for a given 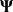, as well as mean phenotype of each group and phenotypic variance.

**Table 1:** Populating of matrix 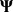 for given interactions.

Figure of occurrence	Simulated interaction	Matrix 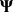
Figure 2	As in Figure 1 A	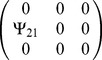
Figure 3	As in Figure 1 B	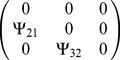
Figure 4, 5, 6, 7	As in Figure 1 C	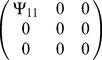

## Supporting Information

Appendix S1 (PDF)
