# Supplementary material for: Phenotypic and Evolutionary Consequences of Social Behaviours: Interactions among Individuals Affect Direct Genetic Effects
Source: PLoS One. 2012 Nov 30;7(11):e46273. doi: 10.1371/journal.pone.0046273 (PMC3511512; doi:10.1371/journal.pone.0046273)
Supplement: Appendix S1 — (PDF) [file pone.0046273.s001.pdf]

Appendix

We begin by generalizing Moore et al.'s model [20] for the case with N individuals

$$\mathbf{p}_i = \mathbf{\Gamma}\mathbf{g}_i + \sum_{j \neq i}^{N-1} \mathbf{\Psi}\mathbf{p}_j + \mathbf{e}_i \quad \text{(A-1)}$$

In our model, $\mathbf{g}_i$ (genotype) denotes a column vector of genes of an individual i, $\mathbf{p}_i$ (phenotype) is a column vector of corresponding traits (same as $\mathbf{Z}$ in [20]). Matrix $\mathbf{\Gamma}$ mediates the translation of individual's own genes $\mathbf{g}_i$ into its trait values. Matrix $\mathbf{\Psi}$ is an interaction matrix, where Ψ_{kl} defines the effect of the partner's trait l on the trait k of the focal individual. For simplification, we do not consider environmental effects other than those caused by social interactions.

We can rewrite equation A-1 as

$$\mathbf{p}_i = \mathbf{\Gamma}\mathbf{g}_i + \sum_{j}^{N} \mathbf{\Psi}\mathbf{p}_j - \mathbf{\Psi}\mathbf{p}_i + \mathbf{e}_i \quad \text{(A-2)}$$

Summation over all individuals in a group yields

$$\sum_{i}^{N} \mathbf{p}_i = \sum_{i}^{N} \mathbf{\Gamma}\mathbf{g}_i + \sum_{i}^{N}\sum_{j}^{N} \mathbf{\Psi}\mathbf{p}_j - \sum_{i}^{N} \mathbf{\Psi}\mathbf{p}_i + \sum_{i}^{N} \mathbf{e}_i$$

$$\sum_{i}^{N} \mathbf{p}_i = \sum_{i}^{N} \mathbf{\Gamma}\mathbf{g}_i + \sum_{i}^{N} N\mathbf{\Psi}\mathbf{p}_i - \sum_{i}^{N} \mathbf{\Psi}\mathbf{p}_i + \sum_{i}^{N} \mathbf{e}_i$$

$$\sum_{i}^{N} \mathbf{p}_i - \sum_{i}^{N} N\mathbf{\Psi}\mathbf{p}_i + \sum_{i}^{N} \mathbf{\Psi}\mathbf{p}_i = \sum_{i}^{N} \mathbf{\Gamma}\mathbf{g}_i + \sum_{i}^{N} \mathbf{e}_i \quad \text{(A-3)}$$

Now, we can express the sum of all phenotypic values as

$$(\mathbf{I} - N\mathbf{\Psi} + \mathbf{\Psi})\sum_{i}^{N} \mathbf{p}_i = \sum_{i}^{N}(\mathbf{\Gamma}\mathbf{g}_i + \mathbf{e}_i)$$

$$\sum_{i}^{N} \mathbf{p}_i = (\mathbf{I} - N\mathbf{\Psi} + \mathbf{\Psi})^{-1}\sum_{i}^{N}(\mathbf{\Gamma}\mathbf{g}_i + \mathbf{e}_i) \quad \text{(A-4)}$$

A substitution of equation A-4 into equation A-2, we are able to express the phenotype of the focal individual as a function of genotypes of all group members

$$\mathbf{p}_i = \mathbf{\Gamma}\mathbf{g}_i + \mathbf{\Psi}(\mathbf{I} - N\mathbf{\Psi} + \mathbf{\Psi})^{-1}\sum_{j}^{N}(\mathbf{\Gamma}\mathbf{g}_j + \mathbf{e}_j) - \mathbf{\Psi}\mathbf{p}_i + \mathbf{e}_i$$

$$\mathbf{p}_i + \mathbf{\Psi}\mathbf{p}_i = \mathbf{\Gamma}\mathbf{g}_i + \mathbf{\Psi}(\mathbf{I} - N\mathbf{\Psi} + \mathbf{\Psi})^{-1}\sum_{j}^{N}(\mathbf{\Gamma}\mathbf{g}_j + \mathbf{e}_j) + \mathbf{e}_i$$

$$(\mathbf{I} + \mathbf{\Psi})\mathbf{p}_i = \mathbf{\Gamma}\mathbf{g}_i + \mathbf{e}_i + \mathbf{\Psi}(\mathbf{I} - N\mathbf{\Psi} + \mathbf{\Psi})^{-1}\sum_{j}^{N}(\mathbf{\Gamma}\mathbf{g}_j + \mathbf{e}_j)$$

$$\mathbf{p}_i = (\mathbf{I}+\mathbf{\Psi})^{-1}(\mathbf{\Gamma}\mathbf{g}_i+\mathbf{e}_i) + (\mathbf{I}+\mathbf{\Psi})^{-1}\mathbf{\Psi}(\mathbf{I}-N\mathbf{\Psi}+\mathbf{\Psi})^{-1}\sum_{j}^{N}(\mathbf{\Gamma}\mathbf{g}_j+\mathbf{e}_j) \tag{A-5}$$

To separate direct genetic effects (effect of individuals own genotype) from IGEs (effect of genotypes of other individuals), we have to subtract the effects of the genes of the focal individual from the second part of the equation A-5.

$$\begin{aligned}\mathbf{p}_i = (\mathbf{I}+\mathbf{\Psi})^{-1}(\mathbf{\Gamma}\mathbf{g}_i+\mathbf{e}_i) &+ (\mathbf{I}+\mathbf{\Psi})^{-1}\mathbf{\Psi}(\mathbf{I}-N\mathbf{\Psi}+\mathbf{\Psi})^{-1}\sum_{j\neq i}^{N-1}(\mathbf{\Gamma}\mathbf{g}_j+\mathbf{e}_j)\\ &+(\mathbf{I}+\mathbf{\Psi})^{-1}\mathbf{\Psi}(\mathbf{I}-N\mathbf{\Psi}+\mathbf{\Psi})^{-1}(\mathbf{\Gamma}\mathbf{g}_i+\mathbf{e}_i)\end{aligned}$$

$$\begin{aligned}\mathbf{p}_i = &\underbrace{(\mathbf{I}+\mathbf{\Psi})^{-1}(\mathbf{I}+\mathbf{\Psi}(\mathbf{I}-N\mathbf{\Psi}+\mathbf{\Psi})^{-1})\mathbf{\Gamma}\mathbf{g}_i}_{DGE} + \underbrace{(\mathbf{I}+\mathbf{\Psi})^{-1}\mathbf{\Psi}(\mathbf{I}-N\mathbf{\Psi}+\mathbf{\Psi})^{-1}\sum_{j\neq i}^{N-1}\mathbf{\Gamma}\mathbf{g}_j}_{IGE} +\\ &(\mathbf{I}+\mathbf{\Psi})^{-1}(\mathbf{I}+\mathbf{\Psi}(\mathbf{I}-N\mathbf{\Psi}+\mathbf{\Psi})^{-1})\mathbf{e}_i + (\mathbf{I}+\mathbf{\Psi})^{-1}\mathbf{\Psi}(\mathbf{I}-N\mathbf{\Psi}+\mathbf{\Psi})^{-1}\sum_{j\neq i}^{N-1}\mathbf{e}_j\end{aligned} \tag{A-6}$$

Now, the first term of equation A-6 depends only on the focal individual's genotype (i.e. DGE) while the second term depends only on the social partners' genotypes (i.e. IGE).

Simulations

We analysed the role of interaction strength and number of individuals for both direct and indirect genetic effects in our model. Unlike previous frameworks, our model was developed with agent based modelling in mind. Equations were derived for individuals, therefore can be directly used in agent based models for calculations of an individual's phenotype, when genotypes are known.

To illustrate the interaction strength effect on intragroup and intergroup phenotypic variance, we simulated M groups of N individuals (Figure 2 C, D; Figures 5, 6 and 7). All simulations in this study were carried out in Matlab R2010a.

We assume that each individual is haploid and has three genes and three traits. Groups were created by assigning each gene for each individual a random value sampled from a standard normal distribution. Then, the mean genotype of the whole population was calculated and subtracted from the genotype of each individual, thus the population mean genetic value of each gene was set to 0.

Figures 2 A, 3 and 4 were created using equation 2.

Matrix $\mathbf{\Psi}$ (3x3) was populated to describe a given interaction (see Table 1). We calculated phenotypes for all individuals for a given $\mathbf{\Psi}$, as well as mean phenotype of each group and phenotypic variance.

Table 1. Populating of matrix $\mathbf{\Psi}$ for given interactions

Figure of occurrence	Simulated interaction	Matrix $\mathbf{\Psi}$
Figure 2	As in Figure 1 A	$\begin{pmatrix} 0 & 0 & 0 \\ \Psi_{21} & 0 & 0 \\ 0 & 0 & 0 \end{pmatrix}$
Figure 3	As in Figure 1 B	$\begin{pmatrix} 0 & 0 & 0 \\ \Psi_{21} & 0 & 0 \\ 0 & \Psi_{32} & 0 \end{pmatrix}$
Figure 4-7	As in Figure 1 C	$\begin{pmatrix} \Psi_{11} & 0 & 0 \\ 0 & 0 & 0 \\ 0 & 0 & 0 \end{pmatrix}$
